# Sonochemical Preparation of Polymer Nanocomposites

**DOI:** 10.3390/molecules140602095

**Published:** 2009-06-10

**Authors:** Ke Zhang, Bong-Jun Park, Fei-Fei Fang, Hyoung Jin Choi

**Affiliations:** Department of Polymer Science and Engineering, Inha University, Incheon 402-751, Korea; E-mails: zk_smallbird@hanmail.net (K.Z.), bbong96@hanmail.net (B-J.K.), feifeifang@hanmail.net (H-J.C.)

**Keywords:** sonochemistry, ultrasonic, carbon nanotube, polymer, nanocomposite

## Abstract

This review covers sonochemical fabrication of polymer nanocomposites. In addition to its application to the synthesis of various polymeric systems, due to its powerful efficiency, sonochemistry has been widely used not only as the assistant of dispersion for nanomaterials such as carbon nanotubes (CNT) and organophillic clay, but also as a special initiator to enhance polymerization for fabrication of polymer nanocomposites with CNT and metallic nanoparticles. Recent developments in the preparation of multi-walled carbon nanotube/polymer nanocomposites with polystyrene and PMMA, magnetic particle/CNT composites and polymer/clay nanocomposites along with their physical characteristics and potential engineering applications will be introduced. Physical characterizations include morphological, thermal, and rheological properties under either an applied electric or magnetic field.

## Introduction

Since the first report on the use of ultrasound to promoting reaction rates [1], the wide applications of sonochemistry in synthesis have attracted a lot of attention, in not only academia, but also in various fields of chemistry, materials science and chemical engineering [2,3,4]. Ultrasound is a kind of sound lying between 20 kHz and 10 MHz which cannot be heard by the human ear, and it has been reported that high-intensity ultrasound could exceed the attractive force of molecules to produce high concentrations of H· and OH· radicals in water [5,6]. Subsequently, sufficient numbers of cavitation bubbles can be formed. These cavitation bubbles absorb vapor or gas from the solvent to expand until they collapse at the maximum volume. According to “hot spot” mechanism, every cavitation bubble exists as a hot spot in the solvent. In addition, an elevated temperature (>5,000K) and high pressures (>1,000 atm) are produced when the bubble implodes. In addition, the physical changes of mass transport, emulsification, surface cleaning and thermal heating can also be induced by ultrasound, especially in the fields of nanomaterials [7] and recently, in environmental science [8].

In this review, among the various areas of the sonochemical science, mainly its effect and application to the synthesis of polymer nanocomposites will be summarized. In the area of polymer science the extreme conditions produced by ultrasound are known to act as a special initiator to allow chemical bonds to break and thus enhance polymerization. For example, inorganic polysilylene polymers are synthesized by the reductive coupling of dichlorosilanes with sodium in toluene above 100 °C, a process which is poorly reproducible and results in polydisperse molecular weight distributions. Kim *et al*. [9] reported the synthesis of polysilylene at ambient temperature in the presence of ultrasound, with its monodisperse high molecular weight (polydispersity: *Mw/Mn*<1.5), in which it can reach high molecular weight at the early stages of polymerization. Polymerization of methyl methacrylate (MMA) [10], styrene [11] and *n*-butyl acrylate [12] under high-intensity ultrasound were also studied. The rate of initiation of MMA [5] was reported to be strongly dependent on the temperature, solvent properties and ultrasonic intensity. Meanwhile, a continuing ultrasound leads to the degradation of polymer chains, resulting in a low molecular weight at the end. It is known that the double bonds of monomer like vinyl monomers in or beside cavitation bubbles rupture to radicals, initiating the polymerization. The degradation of high-molecular-weight poly(methyl methacrylate) (PMMA) occurred with the increase in sonication time, showing that the final molecular weight is inversely proportional to monomer concentration and the 1/2 exponent of the ultrasonic intensity [10].

Regarding its effect on polymer degradation, it has been effectively adopted for rupturing high molecular weight polymer chains, especially different polysaccharides like xanthan gum and guar gum [13,14,15]. Chun and Park [13] applied ultrasonic degradation as the best mean of obtaining polymer fractions of different molecular weight of both anionic xanthan polyelectrolyte and nonionic schizophyllan rodlike biopolymers, and then studied the ionic strength dependence of intrinsic viscosity as a function of molecular weight. Ultrasonicated xanthan gum [14,15,16] and guar gum [17] have been also investigated and adopted as turbulent drag reducers, in which the turbulent drag reduction phenomenon implies that the frictional resistance in turbulent flow can be drastically reduced by the injection of minute amounts of polymeric additive, thus making that polymer solutions undergoing flow in a pipe require a lower pressure drop to maintain the same flow rates [18,19,20,21].

On the other hand, small size (50 nm) and high molecular weight (>10^6^ g/mol) polystyrene (PS) latex particles were prepared under ultrasonic irradiation with a low level of surfactant [11], indicating that the continuous ultrasonication environment around monomer droplets provides sufficient radicals for polymerization, which leads to a small latex particle size. Therefore, compared with conventional polymerization it can be regarded that sonochemical activation offers some attractive features such as low reaction temperatures, faster polymerization rates and higher molecular weight of polymers. Moreover, ultrasonic irradiation in copolymerization has also been widely studied since it is not only allows short reaction times, but it is also easy to handle. In particular, this method is very useful for those copolymers which are difficult to prepare by conventional methods [22,23]. Recently, ultrasound has been more associated with the production of many different types of polymer nanocomposites of various inorganics such as carbon nanotubes (CNTs), clay, inorganic oxides and magnetic particles, which will be covered in this review.

### Polymer-Carbon Nanotube Nanocomposites

Despite extensive investigations on CNTs since their first discovery, dispersing pristine nanotubes uniformly in solvents is known to be difficult due to their high aspect ratio and the intrinsic van der Waals attraction between nanotubes [24,25]. On the other hand, moderated dispersion of nanotubes can be achieved in some organic solvents with the help of ultrasonication. Ultrasonication has also been used in functionalization of CNTs [26,27] and fabrication of polymer/CNTs nanocomposites [28,29]. To improve load transfer of polymer/CNTs nanocomposites by solvent blending method, nanotubes are generally dispersed in a suitable solvent and then a metastable suspension of nanotubes is mixed with the polymers with the aid of ultrasound. Furthermore, the coagulation or solvent casting method was also used to produce polymer/CNTs nanocomposites.

**Figure 1:**
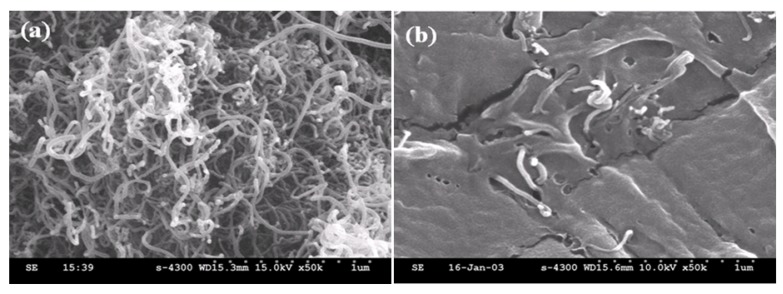
SEM images of (a) acid-treated MWNT and (b) the fracture surface of PMMA/MWNT (0.1wt %) nanocomposites [30].

Park *et al*. [30] reported that PMMA/multi-walled carbon nanotube (MWNT) nanocomposites with different MWNT contents were prepared by *in-situ* bulk polymerization. Initially the acid-treated MWNT was dispersed in a liquid MMA monomer state under ultrasonication (frequency of 28 kHz, power of 600 W). Subsequently, MMA and MWNT-MMA suspension were polymerized in the presence of 2,2-azoisobutyronitrile (AIBN) initiator. Table 1 shows that molecular weight of PMMA increases as the MWNT contents increase due to participation of MWNTs in polymer reaction and the consumption of AIBN [31]. The resultant decrease of AIBN with the addition of MWNTs resulted in the increase in molecular weights of PMMA, and the generation of radicals on the MWNTs’ surface by AIBN triggered the growth of PMMA chains, which then wrapped on the CNT surface [31]. The pristine CNT in Figure 1(a) shows many entangled clusters. After polymerization, most individual MWNTs are well embedded in matrix polymer [Figure 1(b)] and their diameter (about 38 nm) is larger than the pristine one [about 28 nm, Figure 1(a)]. Because well dispersed MWNTs in MMA could be obtained by the aid of ultrasonication and the polymer was shell wrapped around the pristine MWNTs in the subsequent polymerization, it is thought that the PMMA-wrapped MWNTs indicate a good wettability for PMMA matrix.

**Table 1:** Results of GPC analysis for PMMA/ MWNTs prepared via *in-situ* polymerization with and without CNTs [30].

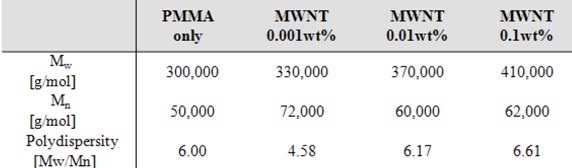

After polymerization, PMMA/MWNT composites were dissolved in chloroform and casted on a Teflon plate to make free standing films. The film obtained by *in-situ* polymerization is relatively transparent and the MWNT aggregations can rarely be seen, indicating that the *in-situ* polymerization method is superior to the post-mixing method for the preparation of MWNT-polymer composites with respect to both MWNT dispersion and optical clarity. 

In addition, MWNT and PS composites were synthesized via an *in-situ* bulk polymerization [32] under the application of ultrasonication without any added initiator. Acid-treated MWNTs were dispersed in styrene monomer by virtue of ultrasound and the radicals generated by decomposition of monomer used to initiate the polymerization. Such an effect of ultrasonication on formation of nanocomposite was focused on investigating its role as an initiator during polymerization in comparison with AIBN.

**Figure 2:**
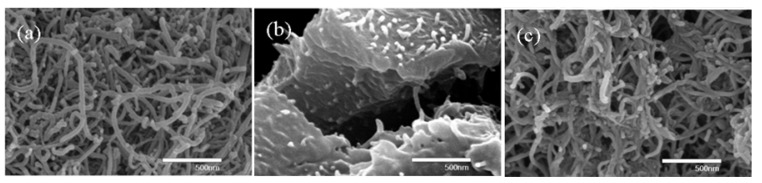
SEM morphology of (a) crude MWNT, (b) fractured surface for PS/MWNTs (2 wt%) composites, (c) PS-g-MWNTs [32].

SEM images of the nanocoposite fracture (Figure 2b) show that well dispersed MWNTs are almost completely embedded in the PS matrix. After removing the un-grafted PS with excess chloroform, the diameter of MWNTs [Figure 2(c)] becomes larger than the pristine one [Figure 2(a)], indicating that the PS is successfully adhered to the MWNTs surface and this combination of PS and MWNTs is quite strong. The wrapped MWNT plays an important role in dispersing the MWNTs (Figure 2b). In addition, the presence of grafted PS on MWNT can also be determined by TGA to 800 °C. Because the weight of pristine MWNT hardly changes to 800 °C, the weight loss appeared near 400 °C in PS-g-MWNTs is attributed to the decomposition of PS [32].

**Figure 3:**
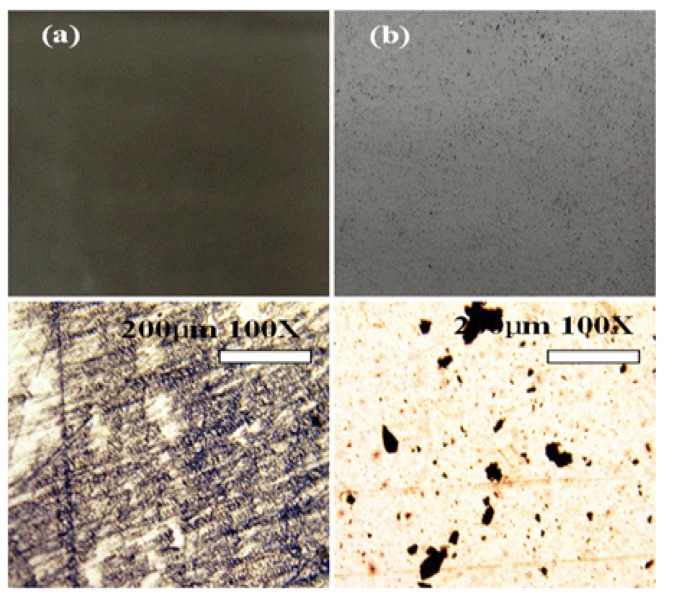
Cast films of (a) PS/MWNT (0.1 wt %) composite by in-situ bulk polymerization and (b) PS /MWNT (0.1 wt %) by solution-mixing for 3h under ultrasonic treatment [32].

Furthermore, from the images of photographs (upper) and optical microscope (lower) from *in-situ* bulk polymerization (Figure 3a) and solution-mixing (Figure 3b), the MWNT in Figure 3(a) is observed to be well dispersed, without any higher opacity agglomeration. However, the film obtained via a solution-mixing processing by directly dispersing MWNTs in PS solution shows undesirable MWNT dispersion [Figure 3(b)], in which the MWNTs aggregate with randomly disjoined different sizes. The dispersion states of CNTs in the polymer matrix directly affect physical properties of the nanocomposites [33,34] which will be enhanced by effective use of their extraordinary properties obtained at the individual nanotube level [35].

**Figure 4:**
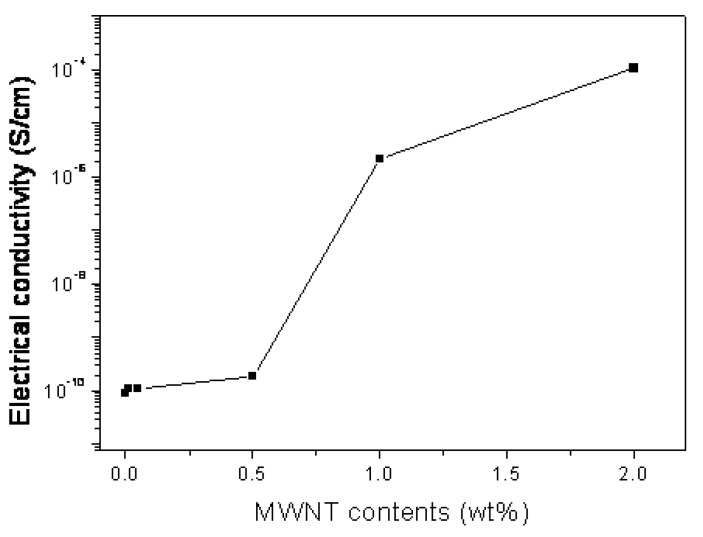
Electrical conductivity vs. MWNT content in the composite [32].

The GPC results on molecular weight for PS/MWNTs composites with and without the initiator, AIBN, indicate that the number-average molecular weight decreased for both cases as the MWNT contents in the composites increased. Gelation time, for the polymer chains which are under the influence of ultrasound, has been increased, due to the increase in the terminal time of polymerization with the addition of MWNT. As a result, the degradation of PS chains caused by ultrasonic treatment increased and the number average molecular weight decreased [36]. In addition, as the MWNT contents increased, the weight-average molecular weights of PS either increased or decreased, depending on the presence or absence of AIBN, respectively. Well dispersed CNTs in the nanocomposites will yield much higher electrical conductivity, even at small loadings because the well dispersed CNTs can effectively provide conductive paths. Figure 4 shows the effect of MWNT content on electrical conductivity, showing that a rapid increase in electrical conductivity takes place when MWNT content exceeds 0.5%. The electrical conductivity of PS/MWNT nanocomposites filled with 1.0wt% MWNT is four orders of magnitude higher than that the composites embedded with 0.5 wt% MWNT. So, a low percolation threshold less than 1.0 wt% CNTs loading is observed in PS/MWNT nanocomposites prepared by *in-situ* bulk polymerization, which is lower than that some composites prepared by other groups [37,38].

**Figure 5:**
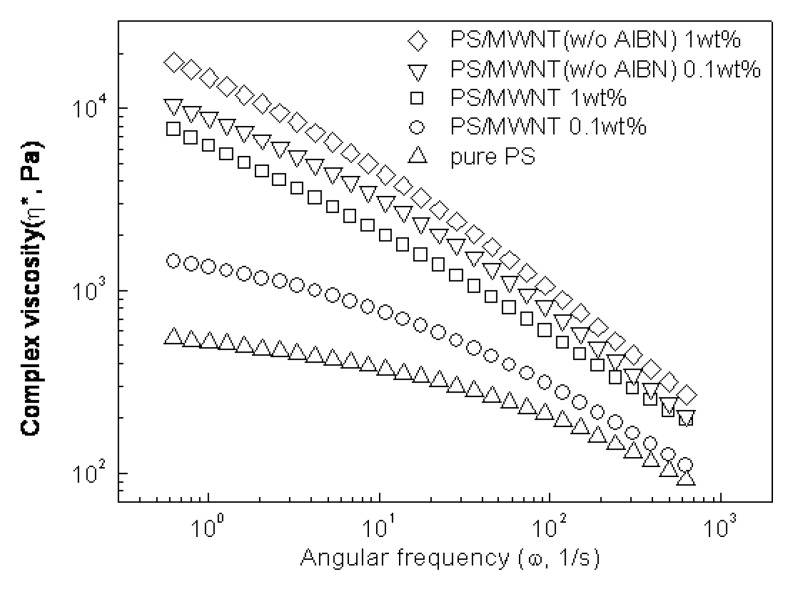
Complex viscosity of PS/MWNT at 230°C [39].

On the other hand, it is well known that information about the rheology of a polymer melt is valuable, because it can be used to analyze molecular structures by branching type and content, entanglement and crosslinking density, along with other valuable rheological parameters. Rheological properties of the PS/MWNT nanocomposites [39] were studied using a rotational rheometer with a parallel plate at a fixed temperature. The effect of initiator (AIBN) on the complex viscosity of nanocomposites as a function of frequency at different MWNT loadings is shown in Figure 5. In the low frequency region, the increase in the complex viscosity is pronounced with the increase of MWNT loadings, and at 0.1 wt% MWNT loading there exists an obvious increment of about one order of magnitude as compared to pure PS in PS/MWNT nanocomposites (without AIBN: w/o AIBN) than in the other, which is thought to be related to the PS molecular weight. Under the same conditions the molecular weight of nanocomposites prepared by ultrasonication irradiation (w/o AIBN) is larger than that fabricated by conventional method (irradiation with AIBN initiator). Generally, the overlapping from different segments of polymer increases with the increment of molecular weight. For high molecular weight polymers, these overlapping-entanglements are not merely lapping between neighboring molecules, so under ultrasonication irradiation, the increment of polymer-polymer interaction in nanocomposites leads to higher complex viscosity at low frequency with more distinct shear thinning behavior over the whole frequency range.

The storage modulus (G´) and loss modulus (G˝) of PS/MWNT nanocomposites (with and without AIBN) with the applied frequency revealed that both G´ and G˝ increase with frequency and MWNT loading. Especially, the increments are pronounced in the low frequency region when MWNT content is increased. However, in the case of poly(vinyl acetate) (PVAc)/MWNT nanocomposites prepared via the solvent casting method [40], the G’ increases with an addition of MWNT loading, compared with that of the PVAc matrix, not as dramatically compared with other polymeric systems. The G” also increases with the MWNT content, while small amount of MWNT was observed to remarkably decrease resistivity of the nanocomposites. This rheological response is similar to the behavior of typical filled-polymer system such as polymer/clay [41,42]. Furthermore, the nanocomposites prepared by ultrasonication irradiation show higher G´ and G˝ relative to the other system with the same MWNT loading at low frequency, suggesting that stronger polymer-polymer, nanotube-polymer or nanotube-nanotube interaction existed.

Like CNTs, carbon black (CB) [43] has been successfully used to trap PVA macro-radicals formed by sonochemical degradation of PVA aqueous solutions to form PVA grafted CB nanocomposites. The PVA grafted CB solution shows better stability after resting than a dispersion system of CB in water or PVA solution. Moreover, it confirmed that PVA grafted CB can improve the compatibility between CB and PVA matrix. 

### Conducting Nanocomposite Particles

Core-shell typed microbeads with MWNT coatings have drawn a lot of attention due not only to their interesting structures, but also their functionalities, especially their electrical conductivity. Either PMMA or PS has been widely used as a core material.

**Figure 6:**
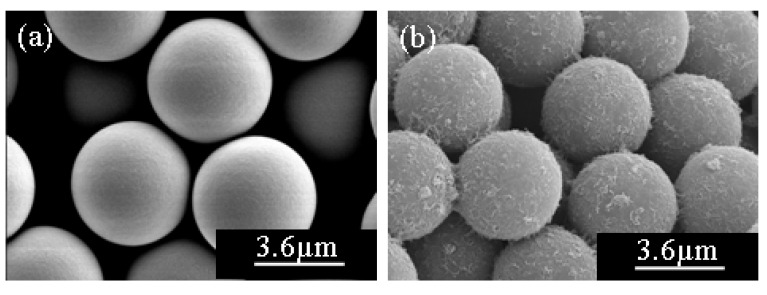
SEM images of carboxylic acid functionalized MWNT –adsorbed onto PMMA microspheres (a) pure PMMA (b) PMMA/ MWNT [44].

Carboxylic acid functionalized MWNTs (4.8 wt %) were successfully adsorbed onto the surface of PMMA microspheres (5 µm) in Di-water under ultrasonic wave [44], in which monodispersed PMMA microspheres were fabricated by dispersion polymerization beforehand and then dispersed into MWNT suspension to form homogeneous colloid solution, forming PMMA/MWNT microspheres. According to the Hansen solubility parameter (
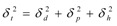
) [45], it is thought that hydrogen bonding component (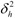) may be the principal factor determining the dispersion states of MWNTs in Di-water, because the nanotubes have carboxylic acid functional groups on the surface of the side wall, which can help to form hydrogen bonding between MWNT and Di-water. Due to the benefit from having carboxylic acid functionalization on the surface of MWNT and the dispersive effect of ultrasonication, more individual nanotubes were well adsorbed onto the PMMA surface instead of aggregated ones (Figure 6). The conductivity of PMMA microspheres is found to be dramatically increased from 1.5×10^-14^ S/cm to 1.4×10^-3^ S/cm after addition of nanotubes. In addition, the ER fluids prepared using PMMA/MWNT microspheres dispersed in silicone oil (10 vol%) exhibit typical ER fibril structure under the applied electrical field of 0.2kV/mm and the structure remained stable as long as the field was applied. It is well known that the superior ER response is also due to the well-dispersed nanotubes on the PMMA surface. The ER fluids are in general suspensions of particles with a higher dielectric constant and/or conductivity than that of the medium oils with a low dielectric constant and a low shear viscosity. They demonstrate a drastic and reversible change in their rheological characteristics under an applied electric field, because the dispersed ER particles are attracted to each other to form fibrillar structure induced by electrostatic attraction of the polarized particles [46,47,48]. Recently, nanoparticles with MWNT and polyaniline synthesized by oxidative dispersion polymerization using PVA as a polymeric stabilizer and HCl as a dopant [49] were reported to exhibit ER characteristics.

### Magnetic Particle Nanocomposites

Ultrasonication has been recently adopted to fabricating CNT-associated magnetic particle nanocomposites. Such magnetic nanocomposites are reported to exhibit magnetoresponsive magnetorheological (MR) phenomena, in which the MR fluids, suspensions of magnetic particles in nonmagnetic medium oil, have drawn much attention due to their controllable rheological characteristics and wide range of engineering applications [50,51]. Carbonyl iron (CI) particles, which are superior species being adopted widely to MR fluids, however, have severe sedimentation problems due to the large mismatch of the particle density to the carrier oil. Therefore, diverse strategies have been introduced to modify pristine CI particles to meet the requirements of industrial application for MR fluids [52,53]. 

Concurrently, CNTs have been extensively studied as filler materials for fabricating polymer/CNT nanocomposites which exhibit synergistic mechanical, electrical, and thermal properties. Functionalization of CNT (COOH/NH_2_-CNT) or physical adsorption of dispersants onto the surface of CNT has been introduced to increase the dispersibility of CNT in solvents [54]. Therefore, considering the self-assembling trend of CNT with reactive groups (COOH/NH_2_-CNT) [52], a dense nest of CNT onto the surface of CI particles was constructed with the aid of using 4-aminobenzoic acid (PABA) as a grafting agent [56]. MR performances of the CI/CNT particles based MR fluids were then analyzed. The PABA was dissolved in distilled-water at 60˚С for 2 h. CI particles were dispersed in this solution and underwent sonication for 15 min to modify their surfaces. The chemically treated CI particles which were washed by DI-water to remove the excess PABA were then added to the reactor, in which a dispersion of COOH-CNT in DI-water was previously added. The reaction was kept under sonication as well as vigorous stirring in order to induce uniform dispersion of the high density CI particles at room temperature. The fabricated CI/CNT particles were separated from the residual CNT solution using a magnet. In order to prepare MR fluids, these CI/CNT particles were dispersed in lubricant oil with a particle concentration of 20 vol%. MR characterization was performed via a rotational rheometer equipped with a magnetorheological device [53].

**Figure 7:**
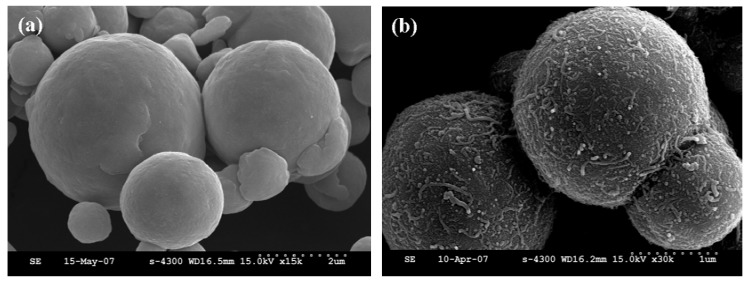
SEM images of pure CI particle (a) and CI/CNT composite particles (b) [56].

Compared with the smooth surface of pure CI particles indicated in Figure 7(a), a considerably rougher surface shown in Figure 7(b) is observed, due to the densely wrapped nest-liked CNTs. In addition, CNTs are randomly spread over the surface without aggregation, demonstrating that the applied sonication not only drives the self-assembling of CNTs but also enhances good dispersion. 

**Figure 8:**
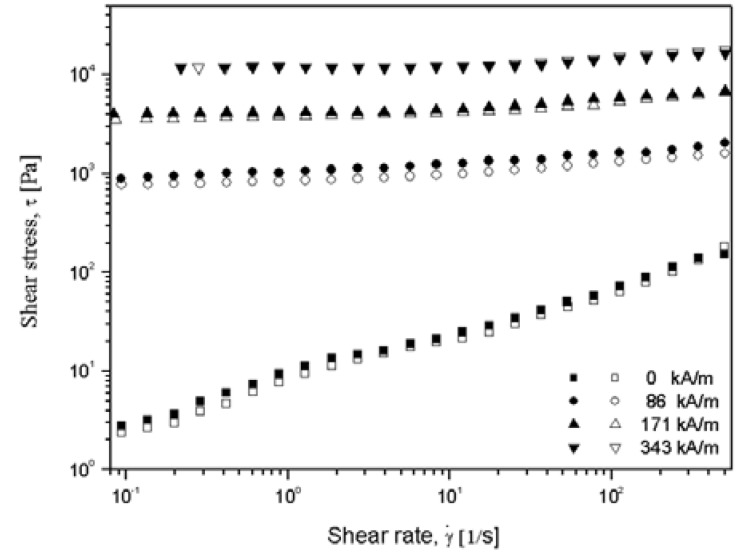
Shear stress as a function of shear rate for both CI/CNT MR fluid (open symbol) and CI MR fluid (closed symbol) under different magnetic field strengths [56].

Figure 8 shows the shear stress as a function of shear rate for both the CI and the CI-CNT based MR fluids under different magnetic field strengths ranging from 0 to 343 kA/m [53]. Typical non-Newtonian fluid behavior without the magnetic field is observed, possibly caused by the remnant magnetization of the magnetic particles [53]. With an applied magnetic field, all of the shear stress curves represent wide plateau range over the whole region of applied shear rate which may be caused by the robustly formed columns due to the strong dipole-dipole interaction among the adjacent magnetic particles. The non-vanishing dynamic yield stress (τ_v_) for steady shear flow which takes crucial part in designing MR devices is defined as the value of shear stress in the limit of zero shear rate. Determining τ_v_ requires an extrapolation of the experimental data at finite shear rates, and the CI/CNT suspension shows the similar τ_v_ as that of the pristine CI suspension which is about 1.61 kPa, 6.7 kPa and 17.8 kPa, corresponding to the strength of magnetic field of 86 kA/m, 171 kA/m and 343 kA/m, respectively. The change of storage modulus and loss modulus under various magnetic field strength was found that both storage and loss moduli do not vary apparently with the growth of angular frequency at a fixed magnetic strength, suggesting that the sample possesses superior solid-like structure rather than liquid-like structure [57]. 

Among other studies, Morel *et al.* [58] have successfully synthesized Fe_3_O_4_@SiO_2_ magnetic nanoparticles (NPs) by an sonochemical approach, finding that the smaller Fe_3_O_4_ NPs with narrow size distribution (4-8 nm) can be achieved under effective ultrasonication. Moreover, the experiment results in the coating of Fe_3_O_4_ NPs with silica indicated that ultrasonication not only accelerated the hydrolysis of TEOS at least 5-fold under the given conditions, but also controlled the thickness of the silica shell in the range of several nanometers. Furthermore, due to the better control of the deposited silica quantities and the rapidity of sonochemical coating, the core-shell Fe_3_O_4_@SiO_2_ NPs prepared by sonochemistry show a higher magnetization value than that for NPs obtained under silent conditions.

### Other Nanocomposite Systems

Polymer/clay nanocomposites have attracted great interest during a last decade due to the enhancement of mechanical and thermal properties as compared to the homopolymer or other fillers. To achieve the optimum dispersion quality and enhance the swelling of layered silicate d-spacing in polymer/clay system, various chemical and mechanical method were utilized, such as clay surface treatment using organic modifiers, utilization of functional polymers, application of different processing conditions, and the use of third component to increase the compatibility. Among these methods, the ultrasonic technique can be used as an effective and convenient method for quality control of the dispersion [59,60], including the dispersion quality of clay in PEO/OMMT nanocomposites with differential fabrication processing [61]. One system was prepared through solvent casting method by the introduction of clay after ultrasonic dispersion into PEO solvent (labeled as PEOC9). Contrary to PEOC9 (sonicated clay dispersion system), non-sonicated clay dispersion was mixed with PEO solution (labeled as NPEOC9), and the rest procedure was the same as with PEOC9. For the MPEOC9 nanocomposites system, the mixture of PEO solution and non-sonicated clay dispersion was sonicated at the end. Finally, BPEO9 was obtained by dispersing OMMT particles directly into PEO solution. In order to get an immiscible PEO-clay blend system, the nanocomposites was achieved by the evaporation of solvent immediately. 

**Figure 9:**
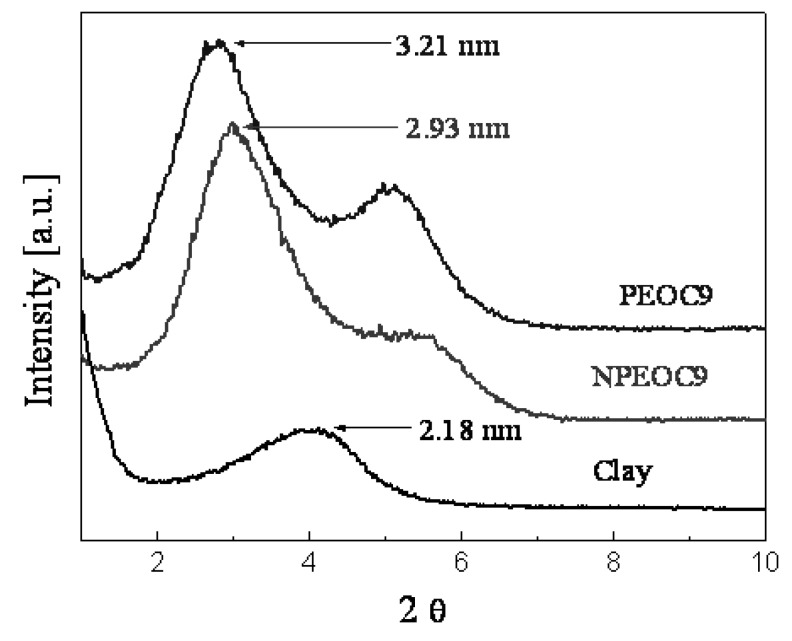
XRD curves of PEO/OMMT nanocomposites with and without sonication for clay dispersion [61].

The interlayer spacing of OMMT (Cloisite 25A) in PEO/OMMT nanocomposites has been determined by the angular position 2θ in XRD pattern (Figure 9) using the Bragg fomula, λ = 2d sin θ (the wavelength (λ) of X-ray is 1.5 Å). In comparison with the pure OMMT, it found that the shift of the diffraction peak toward smaller value of 2θ in the PEO/OMMT nanocomposites. The d_(001)_ expanded from 2.18 nm for pure OMMT to 3.21 nm and 2.93 nm for PEOC9 and NPEOC9, respectively. The nanocomposites which were mixed with sonicated clay dispersion, showed slightly larger interlayer spacing than NPEOC9. Notice that the sonication process plays a role in the dispersion quality and charges in layer spacing. Although more fine dispersion and enhanced swelling were achieved in the sonication step of clay dispersion, the clay particles exist in nanocomposites not only individual platelets but also tactoids. The nanoscopic structures of OMMT in the nanocomposites were observed by TEM, in which some dark ribbons dispersed in PEO matrix, which are considerate as the stacks of organoclay layers. Each clay nanolayer has a lateral dimension of 200 ~ 2,000 nm and a thickness of approximately 1 nm. The stacking of nanolayers forms tactoids (crystalline) that are typically 0.1 ~ 1 μm thick [62]. 

Silicate has excellent barrier properties that prevent permeation of various gases. The addition of clay enhanced the performance by acting as a superior insulator and mass-transport barrier to the volatile products generated during decomposition. Some reports found that a large increase in the onset of decomposition occurs for Polymer/clay nanocomposites as clay content increased [42,63].

**Figure 10:**
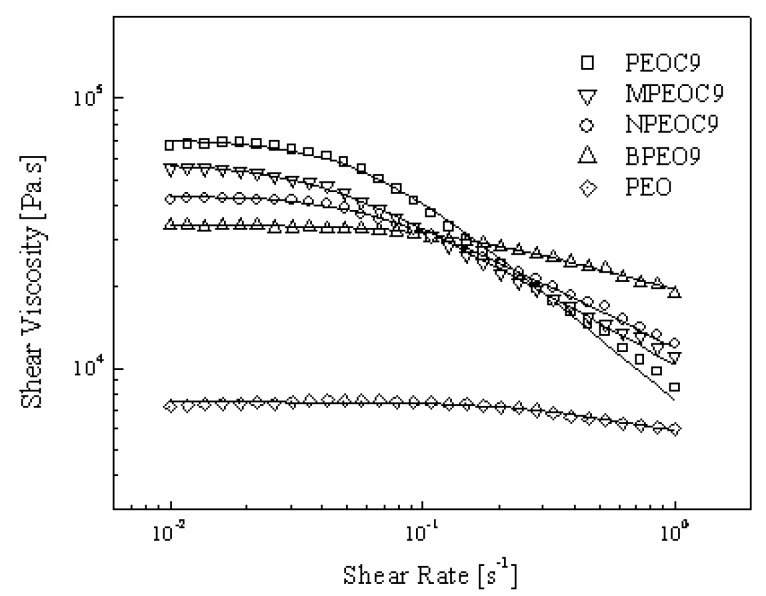
Shear viscosity vs. shear rate for various PEO/OMMT nanocomposites obtained from the different preparation procedures. Symbols represent the experimental data, and solid lines indicate the best fits obtained from Carreau model (Eq. (1)) [61].

In addition, the nanocomposites obtained with different sonication histories showed distinct rheological properties. In Figure 10 PEOC9 exhibited the highest value of steady shear viscosity in lower shear rate region indicating that the most fine clay dispersion was accomplished. On the contrary, the BPEO9 prepared by simple blending of polymer and organoclay showed lowest steady shear viscosity. Thus, the poorest dispersion was obtained without any intercalation and sonification processes. It will be seen from this ultrasonication led to enhancement of swelling of organoclay in polymer/clay nanocomposites to make an ideal state of dispersion. It is clearly that the magnitude of zero shear viscosity is relative to their fabrication processing. Moreover, each system showed varying degree of shear-thinning behavior as the shear rate increases, which is also dependent on the dispersion qualities. PEOC9 with well dispersed organoclay shows more enhanced shear-thinning behavior.

Furthermore, to examine the relationship between shear viscosity (η) and shear rate (*ẏ*), we fit the measured viscosity to the Carreau model [42], given by:

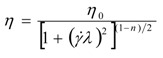
(1)
where, η_0_ is the zero shear rate viscosity, λ is a characteristic (or relaxation) time, and n is a dimensionless parameter. The slope of η *vs*. *ẏ* in the power-law region is (n-1), in which both (1-n) and λ increase with the degree of dispersion, because microstructure transition occur in the nanocomposite from a random to an order orientation in alignment of clay layers under shear flow [64]. It follows that well dispersed organoclay might be difficult to align under flow, which more effectively alter the flow characteristics under shear.

The dynamic moduli of PEO/OMMT nanocomposites as a function of frequency showed that both G′ and G″ increase monotonically for all systems. The decrease in the slope of G′ and G″ at low frequency suggests that nanocomposites present solid-like behavior with introduction of organoclay, and this rheological response is stronger in the nanocomposites which were prepared with sonicated organoclay. ω_c_ cannot be found for PEOC9 due to G′ > G″ for all frequency ranges. It is considered that the tactoids and individual clay layers with mesoscopic structure cannot rotate freely when they are subjected to shear. This incomplete relaxation due to the physical jamming or percolation leads the solid-like behavior observed in both intercalated and exfoliated nanocomposites [65].

Furthermore, Wang *et al*. [66] reported that preparation of MWNT/ZnS core/shell hetero-structured nanocomposites by a facile solution-chemical method and the ZnS shell can be controlled by adjusting the experimental conditions. In addition, with the aid of sonication, they found that the core/shell hetero-structured MWNT/ZnS is easily encapsulated within a uniform SiO_2_ layer using a versatile modified-Stöber process, showing better optical properties and thermal stability.

## Conclusions

The recent development of polymer nanocomposites prepred via ultrasonication with inorganics such as CNT, clay, inorganic oxides and magnetic particles have been reviewed. In these processes the sonochemistry triggers and accelerates chemical reactions with acoustic energy, which effectively shortens reaction time, improves rates of production, reduces reaction condition and achieves some reactions that cannot be accomplished by conventional methods. Utilization of ultrasonication opens up a way for fabrication of various polymer nanocomposites not only for filler dispersion but also polymerization. As a special tool, we anticipate that sonochemistry will be attract extensive attention worldwide for nanocomposite applications.
